# Bioinformatic prediction of potential T cell epitopes for SARS-Cov-2

**DOI:** 10.1038/s10038-020-0771-5

**Published:** 2020-05-06

**Authors:** Kazuma Kiyotani, Yujiro Toyoshima, Kensaku Nemoto, Yusuke Nakamura

**Affiliations:** Project for Immunogenomics, Cancer Precision Medicine Center, Japanese Foundation for Cancer Research, Tokyo, 135-8550 Japan

**Keywords:** Immunogenetics, High-throughput screening

## Abstract

To control and prevent the current COVID-19 pandemic, the development of novel vaccines is an emergent issue. In addition, we need to develop tools that can measure/monitor T-cell and B-cell responses to know how our immune system is responding to this deleterious virus. However, little information is currently available about the immune target epitopes of novel coronavirus (SARS-CoV-2) to induce host immune responses. Through a comprehensive bioinformatic screening of potential epitopes derived from the SARS-CoV-2 sequences for HLAs commonly present in the Japanese population, we identified 2013 and 1399 possible peptide epitopes that are likely to have the high affinity (<0.5%- and 2%-rank, respectively) to HLA class I and II molecules, respectively, that may induce CD8^+^ and CD4^+^ T-cell responses. These epitopes distributed across the structural (spike, envelope, membrane, and nucleocapsid proteins) and the nonstructural proteins (proteins corresponding to six open reading frames); however, we found several regions where high-affinity epitopes were significantly enriched. By comparing the sequences of these predicted T cell epitopes to the other coronaviruses, we identified 781 HLA-class I and 418 HLA-class II epitopes that have high homologies to SARS-CoV. To further select commonly-available epitopes that would be applicable to larger populations, we calculated population coverages based on the allele frequencies of HLA molecules, and found 2 HLA-class I epitopes covering 83.8% of the Japanese population. The findings in the current study provide us valuable information to design widely-available vaccine epitopes against SARS-CoV-2 and also provide the useful information for monitoring T-cell responses.

## Introduction

In December 2019, a cluster of several severe pneumonia cases of unknown etiology was found in the city of Wuhan in Hubei province of China. Shortly thereafter, a novel *Betacoronavirus*, SARS-CoV-2, was identified as a causative microbial agent to cause severe acute respiratory disease. The World Health Organization (WHO) declared the outbreak of a coronavirus disease of 2019 (COVID-19) as public health emergency of international concern and put in place a series of temporary recommendations on January 30. The current outbreak of COVID-19 has nearly 3 million confirmed cases worldwide with more than 200,000 deaths, as of April 27, 2020, according to the WHO. The genome sequences of the SARS-CoV-2 were reported to consist of ~30,000 nucleotides with high sequence similarities to *Betacoronavirus*, including severe acute respiratory syndrome coronavirus (SARS-CoV; 79%) and Middle East respiratory syndrome coronavirus (MERS-CoV; 50%) [1–3]. The SARS-CoV-2 genome, like other coronaviruses, encodes for multiple structural and nonstructural proteins. The structural proteins include spike protein (S), envelope protein (E), membrane glycoprotein (M), nucleocapsid phosphoprotein (N), and the nonstructural proteins include open reading frame 1ab (ORF1ab), ORF3a, ORF6, ORF7a, ORF8, and ORF10. The previous studies have suggested that SARS-CoV-2 has putatively a similar cell entry mechanism and human cell receptor usage [4, 5].

Many researches are now underway to develop effective interventions for controlling and preventing the COVID-19 pandemic, including therapeutic drugs such as inhibitors of the RNA-dependent RNA polymerase or the viral protease, and blockers of virus-cell membrane fusion as well as vaccines, and large scale clinical trials have just begun [6, 7]. For the vaccine design against SARS-CoV-2 and the evaluation of immunogenicity of candidate vaccines, it is important to predict epitopes of SARS-CoV-2 and detect their immune responses to SARS-CoV-2. However, little information is currently available on which parts of the SARS-CoV-2 sequence are important for our immune responses.

Therefore, in the current study, we comprehensively screened potential T cell epitopes from the SARS-CoV-2 sequence using bioinformatic tools, and also assessed the conservation of these epitopes across different coronavirus species, including SARS-CoV and MERS-CoV.

## Methods

### Coronavirus sequences

Full-length viral nucleotide sequences of SARS-CoV-2 (accession number MN908947 and MN996527-MN996531) [1, 2], SARS-CoV (accession number AY274119, AY278488 and AY390556), bat-derived SARS-like coronavirus (bat-SL-CoV) RaTG13 (accession number MN996532), and MERS-CoV (accession number JX869059) were downloaded from the NCBI GenBank.

### Comparison of coronavirus sequences

Alignment of downloaded sequences was done with Genetyx software (version 8.0.0). The similarity among the sequences was visualized using SimPlot software (version 3.5.1) [8], with the consensus sequence of SARS-CoV-2 isolated from Wuhan-Hu-1 (MN908947) as the query.

### T cell epitope prediction for SARS-CoV-2

Epitope prediction was carried out using the predicted proteins, including S, E, N, M, and ORFs (corresponding to accession numbers QHD43415-QHD43423, QHI42199) of the reference SARS-CoV-2_Wuhan-Hu-1 (accession number MN908947). To predict HLA-class I epitopes, we selected 7, 10, 8 of human leukocyte antigen-A (*HLA-A)*, *HLA-B*, *HLA-C* alleles, respectively, which were reported to be present in more than 5% frequencies in the Japanese population (Supplementary Table 1) [9]. For HLA-class II epitope prediction, we selected 5 and 6 haplotypes of *HLA-DPA1-DPB1* and *HLA-DQA1-DQB1*, respectively, and 7 alleles of *HLA-DRB1* that are frequently observed in the Japanese populations (Supplementary Table 1) [9, 10].

Binding affinity to HLA class I molecules was calculated for all 9- and 10-mer peptides from SARS-CoV-2 proteins using NetMHCv4.0 and NetMHCpanv4.0 software [11, 12]. We selected the top 0.5%-ranked epitopes based on the prediction score as strongly binding epitopes. Binding affinity to HLA class II molecules was calculated for all 15-mer peptides from SARS-CoV-2 proteins using NetMHCIIpanv3.1 software [13]. We applied the threshold of top 2%-ranked epitopes based on the prediction score as strong binders.

### Mutation analysis

To identify mutations of SARS-CoV-2, we used a total of 6421 SARS-CoV-2 sequences isolated in different areas, including 587 sequences from Asia, 1918 from North America, 3190 from Europe, and 726 from Oceania regions, which were deposited in the Global Initiative on Sharing Avian Influenza Data as of 18 April 2020. We first aligned each of these SARS-CoV-2 sequences to the reference sequence SARS-CoV-2_Wuhan-Hu-1 (accession number MN908947) using BLAT software [14]. After the alignment, we extracted nucleotide sequences corresponding to individual proteins of SARS-CoV-2, translated them to amino acid sequences, and then compared them to reference amino acid sequences of SARS-CoV-2_Wuhan-Hu-1 (accession numbers QHD43415-QHD43423, QHI42199).

### Statistical analysis

Fisher’s exact test was used to analyze the enrichment of epitopes and differences of mutation rates of SARS-CoV-2 isolated from different areas. Statistical analysis was carried out using the R statistical environment version 3.6.1.

## Results

**Fig. 1:**
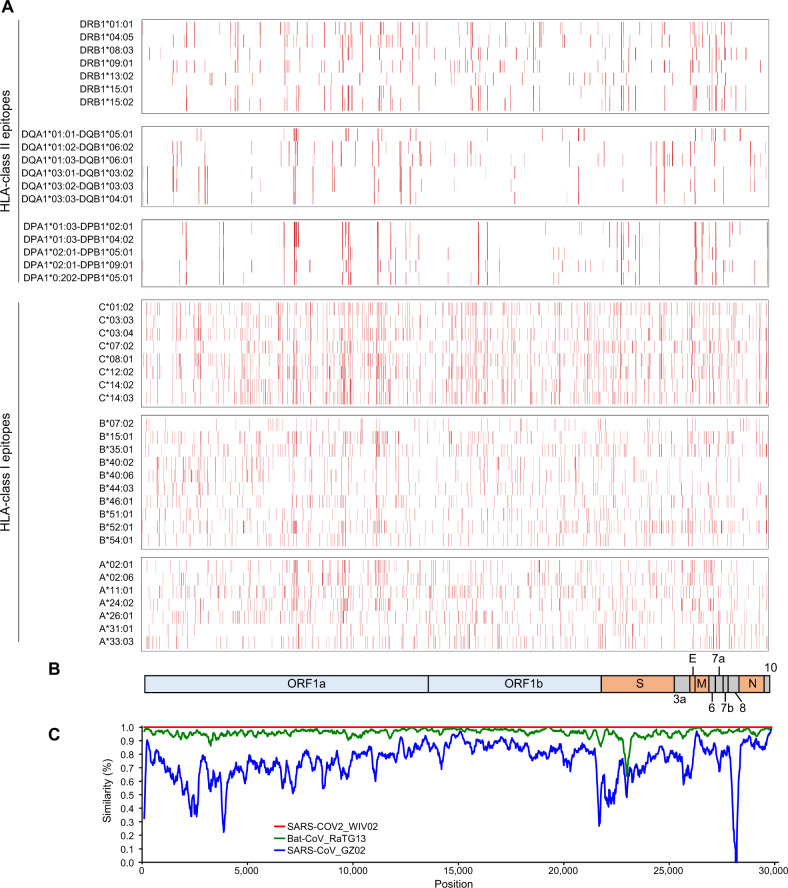
Summary of SARS-CoV-2-dreived T cell epitopes. **a** Distribution of SARS-CoV-2-dreived HLA-class I and II epitopes with the high binding affinity derived from the SARS-CoV-2 protein sequence (SARS-CoV-2_Wuhan-Hu-1) [1]. Red bars represent strong binding affinity epitopes with <0.5% rank and 2% rank, to HLA class I and class II, respectively, for each HLA molecule. **b** Genomic organization of SARS-CoV-2. ORF, open reading frame, S spike, E envelope, M membrane, N nucleocapsid proteins. **c** Similarity plot based on the full-length genome sequence of SARS-CoV-2. Genome sequences of SARS-CoV-2_WIV02 (accession number MN996527), SARS-CoV_GZ02 (AY390556), and Bat-CoV_RaTG13 (MN996532) were compared with SARS-CoV-2_Wuhan-Hu-1 (MN908947)

We first screened potential epitopes that are likely to be presented on certain HLA class I molecules, HLA-A, B, and C molecules, which are commonly observed (frequencies of more than 5%) in the Japanese population [9], using netMHC4.0 and netMHCpan4.0 algorithm [11, 12]. We selected the top 0.5%-ranked (high affinity) peptides derived from the SARS-CoV-2 protein sequences and obtained a total of 2013 unique predicted epitopes (Fig. 1, Table 1 and Supplementary Table 2). The predicted epitopes were significantly enriched in the M protein (*P* = 0.00062, odds ratio = 1.64), whereas less enriched in the N protein (*P* = 0.0074, odds ratio = 0.69). We then performed a screening of HLA-class II-candidate peptide epitopes that show the high affinity to HLA-DPA1, DPB1, DQA1, DQB1, and DRB1, which are common (frequencies of more than 5%) in the Japanese populations [9, 10], using netMHCIIpan3.1 algorithm [13]. We found a total of 1399 possible HLA-class II epitopes after selecting top 2%-ranked peptides (Fig. 1, Table 1 and Supplementary Table 3). The predicted HLA-class II epitopes were enriched in the M protein (*P* = 0.000051, odds ratio = 1.92), ORF3a (*P* = 0.0000016, odds ratio = 2.00), and ORF6 (*P* = 0.000000039, odds ratio = 4.22), whereas less enriched in the N protein (*P* = 0.00031, odds ratio = 0.53).

**Table 1:** SARS-CoV-2-derived T cell epitopes predicted with high affinity to HLA molecules

Protein	Length of protein (amino acids)	Number of epitopes							
		HLA-class I epitopes (rank ≤ 0.5%)				HLA-class II epitopes (rank ≤ 2%)			
		All	HLA-A	HLA-B	HLA-C	All	HLA-DP	HLA-DQ	HLA-DR
ORF1ab	7096	1478	665	937	588	1002	268	377	572
S	1273	248	105	159	110	154	48	54	88
ORF3a	275	69	30	48	28	74	33	25	46
E	75	18	11	11	5	11	3	0	11
M	222	72	36	35	25	57	16	21	35
ORF6	61	8	4	4	2	28	10	12	13
ORF7a	121	26	11	18	9	16	10	0	6
ORF8	121	22	11	14	10	18	4	10	8
N	419	60	21	35	21	32	4	17	12
ORF10	38	12	4	10	7	7	7	0	0
Total		2013	898	1271	805	1399	403	516	791

Since it is reported that SARS-CoV-2 has about 79% and 50% nucleotide sequence homology to SARS-CoV and MERS-CoV, respectively [1–3], we compared the homology of predicted SARS-CoV-2 epitope sequences to 3 SARS-CoVs (BJ01, GZ02, and Tor2) and MERS-CoV to evaluate their cross-reactivities (Table 2 and Supplementary Tables 2 and 3). 781 (38.8%) of the 2013 HLA-class I epitopes are conserved in all the three SARS-CoV sequences. Among them, 633 (81.0%) are located in ORF1ab, and 58 (7.4%), 15 (1.9%), 28 (3.6%), and 33 (4.2%) peptides are located in S, E, M, and N proteins, respectively. 36 (1.8%) of the HLA-class I epitopes show 100% sequence identity to the MERS-CoV protein sequence, and among them, 33 and 3 are located in ORF1ab and S proteins, respectively. Thirty epitopes in ORF1ab are common to both SARS-CoV and MERS-CoV. Among the 1399 possible HLA-class II epitopes, 418 (29.9%) show 100% sequence identity to all the three SARS-CoVs; 362 (86.7%), 40 (11.0%), 4 (1.1%), 4 (1.1%), and 7 (1.9%) are located in ORF1ab, S, E, M, and N proteins, respectively. Ten (2.4%) epitopes, all of which are in ORF1ab, are also conserved in MERS-CoV.

**Table 2:** SARS-CoV-2-derived T cell epitopes common to SARS-CoV or MERS-CoV

Protein	Length of protein (amino acids)	Number of common epitopes to SARS-CoV or MERS-CoV					
		HLA-class I epitopes			HLA-class II epitopes		
		SARS-CoV	MERS-CoV	SARS- and MERS-CoVs	SARS-CoV	MERS-CoV	SARS- and MERS-CoVs
ORF1ab	7096	633	33	30	362	10	9
S	1273	58	3	0	40	0	0
ORF3a	275	4	0	0	0	0	0
E	75	15	0	0	4	0	0
M	222	28	0	0	4	0	0
ORF6	61	0	0	0	0	0	0
ORF7a	121	3	0	0	1	0	0
ORF8	121	7	0	0	0	0	0
N	419	33	0	0	7	0	0
ORF10	38	0	0	0	0	0	0
Total		781	36	30	418	10	9

T cell epitopes, which are likely to be presented commonly on multiple HLA molecules, could cover a larger proportion of individuals/patients. Therefore, we estimated population coverages of SARS-CoV-2-derived, HLA-class I- and II-presented epitopes with high binding affinity on the basis of the allele/haplotype frequencies of *HLA* (Table 3 and Supplementary Tables 4, 5 and 6). Two epitopes in ORF1ab, ORF1ab2168-2176, and ORF1ab4089-4098, that were predicted to have strong affinity to HLA-A*24:02, HLA-A*02:01, and HLA-A*02:06 showed the highest coverage of 83.8% of the Japanese population. ORF1ab2168-2176 was also predicted as an epitope binding to four HLA-C molecules, including HLA-C*01:02, HLA-C*08:01, HLA-C*12:02, and HLA-C*14:02, which cover 76.5% of the Japanese. Two epitopes in S protein, S268-277, and S448-457, covered more than 70% of Japanese. HLA-oligomers with these peptides are also useful for monitoring the CD8^+^ T-cell responses in the patients and silently-infected individuals.

**Table 3:** SARS-CoV-2-derived HLA-class I epitopes with high coverage of Japanese population based on HLA-A frequency

Protein	Position in protein	Peptide length	Peptide sequence	Population coverage^a^					
				HLA-A		HLA-B			HLA-C
ORF1ab	2168	9	YMPYFFTLL	83.8%	A*02:01, A*02:06, A*24:02	0.0%	–	76.5%	C*01:02, C*08:01, C*12:02, C*14:02
ORF1ab	4089	10	FTYASALWEI	83.8%	A*02:01, A*02:06, A*24:02	22.0%	B*52:01	22.2%	C*12:02
ORF1ab	3653	10	VYMPASWVMR	75.7%	A*24:02, A*31:01, A*33:03	0.0%	–	0.0%	–
S	448	10	NYNYLYRLFR	75.7%	A*24:02, A*31:01, A*33:03	0.0%	–	0.0%	–
ORF1ab	3654	10	YMPASWVMRI	75.1%	A*02:01, A*24:02	14.1%	B*51:01	0.0%	–
ORF1ab	1674	10	CYLATALLTL	75.1%	A*02:01, A*24:02	0.0%	–	0.0%	–
ORF1ab	6418	10	LYLDAYNMMI	75.1%	A*02:01, A*24:02	0.0%	–	0.0%	–
S	268	10	GYLQPRTFLL	75.1%	A*02:01, A*24:02	0.0%	–	0.0%	–
ORF3a	106	10	LYLYALVYFL	75.1%	A*02:01, A*24:02	0.0%	–	0.0%	–
ORF1ab	3605	10	FLYENAFLPF	72.3%	A*02:06, A*24:02	24.3%	B*15:01, B*46:01	74.7%	C*03:04, C*07:02, C*08:01, C*12:02, C*14:03
ORF1ab	3126	10	IQWMVMFTPL	72.3%	A*02:06, A*24:02	33.4%	–	0.0%	–

^a^Calculated based on the allele frequency of HLA (Supplementary Table 1)

**Fig. 2:**
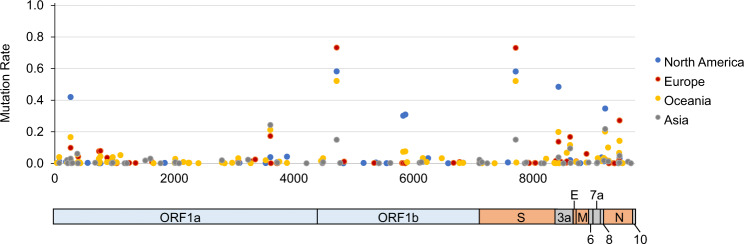
Distribution of mutation rates of SARS-CoV-2. A total of 6421 SARS-CoV-2 sequences isolated from four different regions; 587 viruses from an Asian region, 1918 from a North American region, 3190 from European countries, and 726 from an Oceanian region, were compared with the reference protein sequence of SARS-CoV-2_Wuhan-Hu-1 [1]. 156 amino acid mutations, which were observed at more than 0.5% frequencies in at least one region, were plotted

All replicating viruses, including coronavirus, accumulate some mutations that persist due to natural selection, and these mutations contribute to an escape from immune responses. Thus, we finally investigated mutation rates in 6421 SARS-CoV-2 genome sequences isolated from patients/individuals in four different regions, including Asia, North America, Europe, and Oceania, and identified a total of 156 amino acid mutations, which were observed at more than 0.5% frequencies (Fig. 2, Table 4 and Supplementary Table 7). ORF1ab P4715L and S D614G, which were previously reported [15, 16], were commonly found in all the four regions, although the frequencies of ORF1ab 4715L and S 614G types were significantly lower in Asian countries than other countries (15.4% vs. 52.6–73.7%; *P* = 3.81 × 10^−125^). ORF1ab P5828L and ORF1ab Y5865C were predominant in only North America (30.6% vs. others 0–7.8%; *P* = 3.26 × 10^−237^ and 31.4% vs. 0–8.1%; 5.54 × 10^−246^, respectively). N P13L was frequently observed in only Oceania (10.5% vs. others 0.13–1.7%; *P* = 4.41 × 10^−64^) and N R203K/G204R were observed at higher frequencies in Oceania and Europe compared with the other regions (14.7–27.6% vs. 3.6–5.3%; *P* = 2.56 × 10^−105^). We found no mutation in the epitope sequences described above.

**Table 4:** Mutations frequently (≥10%) observed in SARS-CoV-2 isolated from four different regions

Protein	Position in protein	Reference amino acid^a^	Mutant amino acid	Mutation frequency			
				Asia (*N* = 587)	North America (*N* = 1918)	Europe (*N* = 3190)	Oceania (*N* = 726)
ORF1ab	265	T	I	0.0346	0.424	0.104	0.171
ORF1ab	3606	L	F	0.248	0.0438	0.178	0.17
ORF1ab	4715	P	L	0.154	0.587	0.737	0.526
ORF1ab	5828	P	L	0	0.306	0.00599	0.0783
ORF1ab	5865	Y	C	0	0.314	0.00530	0.0805
S	614	D	G	0.155	0.586	0.736	0.526
ORF3a	57	Q	H	0.0426	0.489	0.142	0.204
ORF3a	251	G	V	0.0991	0.0256	0.172	0.121
ORF8	84	L	S	0.222	0.351	0.0295	0.206
N	13	P	L	0.0171	0.00313	0.00126	0.105
N	203	R	K	0.0532	0.0365	0.276	0.148
N	204	G	R	0.0532	0.0355	0.276	0.147

^a^Protein sequence based on the reference SARS-CoV-2_Wuhan-Hu-1 sequence (GenBank accession number MN908947)

## Discussion

To control the current COVID-19 pandemic and prevent the second pandemic in the near future, the development of new drugs and vaccines, and the establishment of tools investigating the immune responses in patients or silently-infected individuals are urgent issues. Especially, effective vaccination or immunotherapy could play a significant role in suppressing the spread of the virus. Since antibodies recognize cell surface proteins, the targets of antibody-based vaccine were limited. Upon viral infection, the viral proteins express in the infected cells and are processed into small peptides by proteasomes. These peptides are then presented by HLA molecules on the surface of the infected cells and recognized by T cells through their T cell receptors. Thus, the potential T cell epitopes can be derived from any of the viral structural and nonstructural proteins. In this study, using the bioinformatics tools, we comprehensively screened potential SARS-CoV-2-derived, HLA-class I- and II-presented epitopes for 43 *HLA* alleles that are common in the Japanese population, and identified 2013 and 1399 epitopes, respectively. 781 HLA-class I and 418 HLA-class II epitopes are considered to be common between SARS-CoV-2 and SARS-CoV (Table 2). We found that four epitopes, S1060-1068, S1220-1229, N222-230, and N315-324 of SARS-CoV-2, have exactly same sequences reported as immunogenic SARS-CoV-derived epitopes for HLA-A*02:01, that correspond to S1042-1050, S1203-1211, N223-231, and N317-325 of SARS-CoV, respectively [17, 18]. Interestingly, SARS-CoV-2-derived S1060-1068 epitope is also predicted as high affinity epitopes for HLA-A*02:06 (belonging to the same HLA-A*02 family), HLA-B*52:01, and HLA-C*12:02. In the Japanese population, HLA-A*24:02 is the most common HLA-A molecule with the allelic frequency of 37.8% (implying that 61.3% of the Japanese have at least one HLA-A*24:02 allele), followed by 12.3% and 9.6% of HLA-A*02:01 and HLA-A*02:06, respectively. Two epitopes in ORF1ab, ORF1ab2168-2176, and ORF1ab4089-4098, latter of which is conserved in SARS-CoV, were predicted to have the strong affinity to HLA-A*24:02 as well as HLA-A*02:01 and HLA-A*02:06. Based on their allele frequency, these epitopes could cover 83.8% of the Japanese individuals. Since no mutation was identified in these epitope sequences we identified, these potential candidate epitopes could lead to the contribution to development of rationally designed epitope-based peptide vaccines against SARS-CoV-2. If these epitopes are immunogenic, we are able to use HLA-oligomer with each of these peptides for monitoring T-cell responses in patients and silently-infected individuals. In addition, several reports have suggested that a subset of patients with severe COVID-19 might have a cytokine release syndrome [19, 20]. These HLA-oligomers might be useful to predict and monitor acute T-cell responses in COVID-19 patients who cause these life-threatning symptoms.

In conclusion, through bioinformatic screening, we identified a large number of potential T cell epitopes, some of which could cover other coronavirus spices, including SARS-CoV. These peptides can possibly cover a large proportion of the Japanese population. Although further experimental proof to evaluate immunogenicity of the predicted peptides is required, we hope our findings in the current study could contribute to designing vaccines (DNA or RNA vaccine, inactivated viral vaccines, or peptides vaccines) and evaluating these vaccines, and also to immune monitoring of SARS-CoV2-infected patients.

## Supplementary information

Supplementary Table 1

Supplementary Table 2

Supplementary Table 3

Supplementary Table 4

Supplementary Table 5

Supplementary Table 6

Supplementary Table 7
